# Nutraceuticals as Modulators of Molecular Placental Pathways: Their Potential to Prevent and Support the Treatment of Preeclampsia

**DOI:** 10.3390/ijms252212167

**Published:** 2024-11-13

**Authors:** Patrycja Bukowska, Michalina Bralewska, Tadeusz Pietrucha, Agata Sakowicz

**Affiliations:** Department of Medical Biotechnology, Medical University of Lodz, Zeligowskiego 7/9, 90-752 Lodz, Poland

**Keywords:** preeclampsia, placental molecular pathways, plant compounds, plant extracts, probiotics

## Abstract

Preeclampsia (PE) is a serious condition characterized by new-onset hypertension and proteinuria or organ dysfunction after the 20th week of gestation, making it a leading cause of maternal and fetal mortality worldwide. Despite extensive research, significant gaps remain in understanding the mechanisms underlying PE, contributing to the ineffectiveness of current prevention and treatment strategies. Consequently, premature cesarean sections often become the primary intervention to safeguard maternal and fetal health. Emerging evidence indicates that placental insufficiency, driven by molecular disturbances, plays a central role in the development of PE. Additionally, the maternal microbiome may be implicated in the pathomechanism of preeclampsia by secreting metabolites that influence maternal inflammation and oxidative stress, thereby affecting placental health. Given the limitations of pharmaceuticals during pregnancy due to potential risks to fetal development and concerns about teratogenic effects, nutraceuticals may provide safer alternatives. Nutraceuticals are food products or dietary supplements that offer health benefits beyond basic nutrition, including plant extracts or probiotics. Their historical use in traditional medicine has provided valuable insights into their safety and efficacy, including for pregnant women. This review will examine how the adoption of nutraceuticals can enhance dysregulated placental pathways, potentially offering benefits in the prevention and treatment of preeclampsia.

## 1. Introduction

Preeclampsia (PE) is a pregnancy-specific disorder that typically manifests after the 20th week of gestation, characterized by new-onset hypertension (≥140/90 mmHg) and often accompanied by proteinuria. However, the presence of elevated protein levels in urine is not a prerequisite for diagnosis. Hypertension in previously normotensive pregnant women, along with other clinical indicators such as thrombocytopenia, elevated creatinine levels, liver dysfunction, neurological symptoms, or fetal growth restriction, is sufficient for a diagnosis of preeclampsia according to various obstetric and gynecological societies [1,2].

Despite the efforts of numerous scientific teams worldwide to elucidate the pathomechanism of preeclampsia, it remains incompletely understood. As a result, effective methods for prevention and treatment have yet to be established. Consequently, premature delivery remains the only viable option to safeguard both the mother and her child from the severe and potentially fatal consequences of this condition. Therefore, the search for new solutions to enhance maternal–fetal care and mitigate the risks associated with preeclampsia remains a pressing topic in obstetric research. Numerous approaches are being explored, each targeting different aspects of the condition. Some strategies focus specifically on improving maternal blood pressure, while others aim to eliminate placental factors circulating in the maternal bloodstream that disrupt endothelial function. Additionally, some methods concentrate on reducing maternal inflammation [3]. Among these approaches, several focus on enhancing placental function and development, as it is evident that this organ plays a central role in the pathophysiology of preeclampsia.

Placental dysfunction originates early in gestation, i.e., weeks before the onset of preeclampsia, and is linked to inadequate trophoblast invasion into maternal uterine vessels. This shallow invasion results in the inadequate remodeling of the uterine vessels into wider, low-resistance structures, which impairs the delivery of oxygen and nutrients to the developing placenta and fetus. Placental dysfunction manifests at multiple levels, with numerous studies highlighting disturbances in the anatomical structure of the placenta. In humans, placental pathology associated with preeclampsia is characterized by features such as decidual arteriopathy, which includes acute atherosis, fibrinoid necrosis, and the incomplete or absent physiological remodeling of spiral arteries. Additionally, distal villous hypoplasia and increased syncytial knots are commonly observed [4,5]. In animal models, especially rodent models of preeclampsia, similar pathological changes are observed. For instance, extensive vascular damage is evident, including the presence of fibrin thrombi within blood vessel lumens. The labyrinth layer—critical for maternal–fetal gas and nutrient exchange—exhibits disorganized structures. Furthermore, the placentas obtained from these models, similar to those found in human preeclampsia, often exhibit fibrous deposits and infiltration by inflammatory cells, including macrophages and lymphocytes. This pattern suggests the presence of an active inflammatory response [6,7].

The histological alterations observed in placental tissues are closely linked to placental dysfunction, characterized by increased secretion of preeclampsia-associated factors such as soluble fms-like tyrosine kinase-1 (sFlt-1) and soluble endoglin (sENG). Concurrently, there is a significant reduction in proangiogenic factors, including placental growth factor (PlGF) and vascular endothelial growth factor (VEGF) [8,9]. All these changes indicate a dysregulation of the molecular processes occurring within placental cells. While not all molecular pathways implicated in preeclampsia have been fully identified, it is crucial to consider those currently recognized in the pathomechanism of the condition when developing new therapeutic strategies.

Consequently, it is reasonable to hypothesize that inhibiting these dysregulated pathways or enhancing their activity through novel therapeutic agents may provide protective benefits for mothers against the onset of preeclampsia. Additionally, such interventions could improve maternal health outcomes in cases where preeclampsia has already developed. Given that pregnancy is a critical period during which not all therapeutic substances are permissible due to potential side effects on the developing fetus, the selection of potential anti-preeclamptic agents becomes particularly challenging. However, natural products present a promising alternative. Their long history of use in treating various diseases supports their safety profile, making them appealing candidates for further investigation [10]. The extensive application of natural products in traditional medicine underscores their potential as safer therapeutic options, particularly in sensitive populations such as pregnant women.

This review article investigates the therapeutic potential of natural products, particularly focusing on plant extracts and their active compounds, for the prevention and treatment of preeclampsia through the modulation of molecular processes that are dysregulated in preeclamptic placentas. Additionally, this manuscript delves into the maternal microbiome as a vital source of natural products, examining its role in sustaining healthy gestation. Strategies for restoring a balanced maternal microflora will be thoroughly discussed as a promising approach to prevent and treat preeclampsia, highlighting the multifaceted relationship between microbial health and pregnancy outcomes.

## 2. The Placenta and Its Molecular Pathways in Healthy and Preeclamptic Gestations

### 2.1. NFκB Activation Pathways

A substantial body of evidence emphasizes that the high activity of nuclear factor kappa B (NFκB) characterizes the entire period of preeclamptic gestation, not just its beginning, as is typical for healthy pregnancies. The level of this transcription factor starts to increase in the middle of the menstrual cycle, i.e., before pregnancy, and this increasing tendency accompanies the process of placentation, to be gradually silenced with the beginning of the second trimester of uncomplicated gestation [11,12]. In preeclampsia, this reduction in NFκB activity does not appear, and it is recognized as a one of the main reasons of inflammation being observed on the maternal–fetal contact surface and in the maternal blood. Indeed, NFκB is recognized for the regulation of the transcriptional activity of over 400 genes, including those strongly related to inflammation (e.g., tumor necrosis factor alpha -TNFα, interleukin 1-IL-1, IL-6, and IL-8, or cyclooxygenase—COX2), for which elevated levels are a trailer of preeclampsia development [3].

Although the high activity of NFκB is a hallmark of preeclampsia, little is known about the molecular pathways that are responsible for the activation of this transcription factor at each stage of both uncomplicated and complicated gestations. Some pieces of evidence obtained based on the animal studies suggest that the beginning of pregnancy is related to the alternative mechanism of the activation of NFκB, i.e., dependent on the NF-κB-inducing kinase/inhibitory-κB kinase (NIK-IKKα) complex [11]. This mechanism is typical for the first trimester decidua, but it is unknown whether it is also valid in the trophoblastic cells at this stage of gestation. It is also unknown whether this mechanism differs between first trimesters of uncomplicated and complicated-by-preeclampsia gestation. This divergence in the machinery of NFκB activation between healthy and preeclampsia-complicated gestations may exist, as more recent studies suggest that the launching pathway of NFκB activation characteristic for placental cells living in the preeclamptic environment is driven by the tumor protein p53/ribosomal protein S6 kinase alpha-1 (p53/RSK1) complex [13]. This mechanism is independent of the cytoplasmic degradation of the NFκB inhibitor, IκBα, whose inhibitory effect is abolished not by its proteasome degradation but rather by its nuclear dissociation, from NFκB, and recovery to cytoplasm. The activation of this specific preeclampsia mechanism of the activation of NFκB might result in the dysregulation of two other molecular pathways in preeclamptic placentas, i.e., phosphatidylinositide 3-kinase protein kinase B (PI3K/Akt) and mitogen-activated protein kinase/extracellular signal-regulated kinase (MAPK/ERK), known as the ERK1/ERK2 pathway. Both pathways are implicated in the process of the regulation of NFκB activity dependence from its activators, i.e., IKKα and IKKβ, and its inhibitor IκBα [14,15].

### 2.2. PI3K/Akt Signaling Pathway

PI3K and its downstream target, Akt, play crucial roles in various processes essential for the proper functioning of trophoblastic cells [16]. However, the effects of PI3K/Akt pathway activation are contingent upon the specific target proteins located further along this pathway. Notably, the migration and invasion of extravillous trophoblastic cells (EVTs) into maternal uterine vessels—processes that are dysregulated in preeclamptic gestation—are influenced by the downstream regulator of the PI3K/Akt pathway, namely, mTOR (mammalian target of rapamycin).

#### 2.2.1. PI3K/Akt/mTOR Signaling Pathway

The mTOR complex exists in two forms: mTORC1 (known as Raptor) and mTORC2 (known as Rictor) [17]. This PI3K/Akt/mTOR signaling acts by its effectors, including p70 ribosomal protein S6 kinase 1 (S6K1) and eukaryotic translation initiation factor 4E-binding protein 1 (4E-BP1); both are critical for trophoblast function and placental development [18]. S6K1 is essential for protein synthesis and cell growth, promoting trophoblast migration probably through the remodeling of actin filaments to form lamellipodia and filopodia, i.e., structures necessary for cell migration [19]. Conversely, 4E-BP1 acts as a negative regulator of translation initiation, preventing the recitation of mRNA to ribosomes. The phosphorylation of 4E-BP1 by mTORC1 blocks the function of 4E-BP1 and facilitates the translation of mRNAs coding for proteins including those vital for trophoblast function and the growth of the fetus, as was observed in an vitro study on BeWo trophoblastic cells [18]. Moreover, mTOR complex 1 regulates the activity of matrix enzymes such as metalloproteinase 2 and 9, which, by the degradation of the extracellular matrix (ECM), support the process of trophoblast invasion. The lack of functional properties of mTORC1 negatively influences the regulation of ECM degradation as well as modification of process of migration and invasive behavior of extravillous trophoblast cells [17]. Considering the established role of the PI3K/Akt/mTOR axis in trophoblast invasion, migration, and thus, maternal vessel transformation, it is highly likely that this pathway is downregulated in preeclampsia. Indeed, analyses of human placental samples consistently demonstrate inhibition of the PI3K/Akt/mTOR pathway in preeclamptic placentas [20]. Additionally, these findings are reinforced by in vitro research involving the HTR8/SVneo trophoblastic cell line, as well as by in vivo studies using animal models that exhibit features of preeclampsia [21,22,23,24].

#### 2.2.2. PI3K/Akt/eNOS Signaling Pathway

Trophoblast invasion and migration are not the only mechanisms involved in maternal vessel transformation. During the first trimester, EVTs invade the decidua and migrate retrograde into the spiral arteries. As trophoblasts invade, they replace the endothelial lining of the spiral arteries, leading to a loss of the normal muscular structure and the formation of a fibrinoid matrix in which trophoblasts are embedded [25,26]. The transformation of maternal vessels relies on the local production of nitric oxide (NO), a critical molecule that, as supported by numerous studies, is also essential for angiogenesis and, consequently, for proper placental development [27]. Immunocytochemistry studies on human placenta indicate that both cytotrophoblastic cells, particularly extravillous trophoblastic cells [28], and syncytiotrophoblastic cells from the first trimester express nitric oxide synthases (NOS) [29,30,31], including endothelial NOS (eNOS), which catalyzes the oxidation of L-arginine to nitric oxide (NO) and L-citrulline [32]. The activation of eNOS is regulated by the PI3K/Akt/eNOS pathway; indeed, Akt-deficient mice exhibit significant placental hypotrophy, reduced vascularization, and decreased eNOS levels, leading to placental insufficiency, fetal growth restriction, and neonatal mortality [33]. Similarly, placental biopsies obtained from preeclamptic women reveal downregulation of the PI3K/Akt pathway and its downstream element, eNOS [34], compared to control tissues. This downregulation underscores the critical role of the PI3K/Akt/eNOS signaling pathway in maintaining placental health and function. It is also possible that this pathway is involved in the regulation of placental survival, apoptosis, and oxidative stress; however, this hypothesis primarily arises from studies conducted on endothelial cells rather than direct analyses of placental samples [35,36]. Nevertheless, in preeclampsia, both placental apoptosis and oxidative stress are significantly exacerbated, suggesting a disruption in the protective mechanisms typically governed by the PI3K/Akt/eNOS pathway or, potentially, by another pathway, such as PI3K/Akt/MDM2/p53.

#### 2.2.3. PI3K/Akt/MDM2/p53 Signaling Pathway

In this PI3K/Akt/MDM2/p53 signaling axis, Akt mediates the phosphorylation of mouse double minute 2 homolog (MDM2), enhancing its stability and supporting its role in degrading p53 protein levels. This regulation is crucial for preventing cellular senescence in response to oxidative stress [37]. Consequently, when the PI3K/Akt pathway is downregulated, MDM2 may lose its ability to effectively regulate p53, resulting in an accumulation of p53 protein within the cells. This accumulation is critical because p53 serves as a central regulator of cellular apoptosis. Therefore, the imbalance between MDM2 and p53 likely contributes to the increased apoptosis of trophoblastic cells, as demonstrated in fresh placental villous tissue collected from preeclamptic cases compared to non-complicated controls [38].

#### 2.2.4. PI3K/Akt/FOXO3 Signaling Pathway

This apoptotic process may also be influenced by forkhead box O3 (FOXO3), a member of the forkhead transcription factor subfamily. The PI3K/Akt pathway is known for its inhibitory effect on FOXO3 activity through the PI3K/Akt/FOXO3 signaling axis [39]. However, under stressful conditions for trophoblastic cells, such as the hypoxia prevalent in preeclamptic placental tissues, FOXO3 can activate the expression of pro-apoptotic proteins like B-cell lymphoma 2 (Bcl-2)-like protein 11 (BIM) and Bcl-2-associated X protein (Bax) [40,41]. These proteins cooperate with p53 in the intrinsic apoptosis pathway [42].

Although the PI3K/Akt pathway regulates numerous processes essential for proper trophoblast behavior and placental development, it also interacts with other placental pathways. Some of these pathways, such as the MAPK/ERK pathway, work in concert with PI3K/Akt to support similar functions. In contrast, pathways like c-Jun N-terminal kinases (JNK) and p38/MAPK seems to regulate opposing processes.

### 2.3. MAPK Signaling Pathways

The MAPK module consists of three key components: MAPK kinase kinase (MAPKKK), MAPK kinase (MAPKK known as MAP2K, MEK, or MKK), and MAPK. Various stimuli, including growth factors, cytokines, differentiation signals, and stress factors, activate MAPKKK, which then transmits the signal downstream to MAPKK. This process culminates in the phosphorylation of a conserved dual-phosphorylation domain (Thr-Xxx-Tyr) found in four distinct families of MAPKs: ERK1/2, ERK5, JNK1/2/3, and p38α/β/γ/δ [43].

#### 2.3.1. ERK1/2 Pathway

The MAPK/ERK cascade, known as ERK1/2, is the first identified mammalian MAPK pathway. This cascade involves dual-specificity kinases, specifically, MEK1 and MEK2 (also referred to as MKK1 and MKK2), which are responsible for the phosphorylation and activation of ERK1 and ERK2. Upon activation, phosphorylated ERK1/2 dissociate from their cytoplasmic anchors, allowing a significant portion of ERK1/2 to translocate into the nucleus. There, they phosphorylate various transcription factors, including ETS transcription factor ELK1 (Elk1), Fos proto-oncogene (c-Fos), and Jun proto-oncogene (c-Jun), thereby regulating gene expression. Meanwhile, the fraction of ERK1/2 that remains anchored in the cytoplasm can be distributed among various cytoplasmic compartments and organelles [15,44,45]. Cytoplasmic ERK1/2 also influences numerous substrates, with p90 RSK and IKKα and IKKβ being among the most notable. The phosphorylated RSK1 translocates to the nucleus, where it regulates genes involved in cell survival and proliferation [46]. Meanwhile, IκB kinases play a critical role in phosphorylating the NFκB inhibitor IκBα, leading to its proteasomal degradation. This degradation facilitates the migration of NFκB into the nucleus, allowing it to bind to specific elements on DNA and activate target gene expression.

In the context of placental development, phosphorylated ERK1/2 is present in villous cytotrophoblastic cells up to week 12 of human gestation, indicating that this pathway plays a vital role in the proliferation and invasion of these cells [47,48]. Furthermore, animal studies highlight the crucial role of the ERK/MAPK cascade in placental formation and the establishment of the maternal–fetal exchange barrier for gases and nutrients [49,50]. Mice genetically modified to disrupt this pathway exhibit a reduced expression of VEGF, decreased placental vascularization, and increased apoptosis [50]. Additionally, the ERK1/2 pathway regulates the differentiation of placental cells; however, this mechanism is also supported by another subfamily of MAPKs, specifically, p38 kinase, as demonstrated in research on primary cultures of human trophoblasts [48]. The dysregulation of these processes is evident in preeclampsia, suggesting that the ERK1/2 pathway may also be affected. This hypothesis is reinforced by observations from preeclamptic placentas obtained from both human and animal models, as well as trophoblastic cells cultured under conditions that mimic preeclampsia [51,52,53].

#### 2.3.2. ERK5 Pathway

Little is known about the ERK5 pathway in the context of placental development, and there is a significant lack of data regarding its role in the progression of preeclampsia. This kinase is phosphorylated by its upstream regulator, MKK5 (known as MEK5), which is activated by MAPKs, specifically, MEKK2 and MEKK3. Notably, ERK5 possesses a unique ability among the MAPK family to undergo autophosphorylation, enabling it to directly regulate gene transcription [54]. While the downstream substrates regulated by ERK5 are not fully characterized, strong candidates include RSK, gap junction protein Cx43 (connexin-43), and the myocyte enhancer factor-2 (MEF2) transcription factor [54]. All of these proteins have been implicated in gestational development. Little is known about the RSK protein in pregnancy; however, studies not directly related to gestation indicate that RSK regulates the expression of genes whose translational products significantly impact pregnancy development. Key targets include cAMP response element-binding protein (CREB), estrogen receptor-α (ERα), IκBα/NFκB, and c-Fos. Additionally, RSK phosphorylates glycogen synthase kinase-3 (GSK3) [55], which plays an important role in regulating glycogenesis in placental cells. Aberrant glycogen storage has been linked to pregnancy disorders such as diabetes mellitus, fetal growth retardation, and preeclampsia [56]. Cx43 mediates pregnancy-adapted changes in vasodilatory signaling, thereby facilitating endothelial function during pregnancy [57]. In contrast, MEF2 regulates cellular proliferation, differentiation, and invasion across various cell types and tissues, indicating that it may also play a significant role in trophoblast proliferation and differentiation during human placental development. This assertion is supported by in vitro studies involving human cytotrophoblasts isolated from term placentas, which demonstrate that MEF2 influences key processes essential for proper placental function [58,59]. Given that both endothelial adaptation and placental development are dysregulated in preeclampsia, it is plausible that the ERK5 pathway is also downregulated in this condition. This hypothesis is supported by evidence indicating that ERK5 knockout models exhibit embryonic lethality due to placental defects [60].

#### 2.3.3. p38 MAPK Pathway

The p38 MAPK cascade is initiated by several MAPKKKs, including MEKK1-4, in response to chemical or oxidative stress, inflammatory cytokines such as IL-1, IL-6, and TNFα, or microbial infection [61]. These kinases phosphorylate downstream substrates, specifically, the MAPKKs, i.e., MEK3 and MEK6 (also known as MKK3 and MKK6), which exhibit a high degree of specificity toward the p38 protein family, consisting of four isoforms: α, β, γ, and δ [61,62]. Upon activation, p38 MAPK is primarily translocated into the nucleus, where it enhances the transcriptional activity of various transcription factors, including but not limited to activating transcription factor 2 (ATF2), ETS transcription factor ELK1 (Elk-1), c-Fos, c-Jun, peroxisome proliferator activated receptor alpha (PPARα), and signal transducer and activator of transcription (STAT) [63]. Additionally, p38 MAPK influences the stability of the p53 protein, thereby promoting apoptosis, and facilitates the nuclear relocalization of FOXO3 [63]. The behavior of p38 MAPK is closely related to its distribution between nuclear and cytoplasmic compartments following activation. Notably, depending on the stimuli, p38 MAPK does not always translocate to the nucleus; it can also remain in the cytoplasm of activated cells [62]. This cytoplasmic localization allows p38 to regulate various proteins, including kinases, cell death regulators, and membrane receptors [63].

Due to the pleiotropic nature of the p38 pathway, its impact on pregnancy and placental development is multifaceted. This pathway appears to be essential at the onset of gestation for proper blastocyst development, where p38 MAPK activity is required to regulate ribosome-related gene expression, rRNA precursor processing, polysome formation, and protein translation [64]. Additionally, p38 MAPK is involved in the differentiation of trophoblasts in human term placentas, as discussed in relation to the ERK1/2 pathway [48]. However, the excessive activation of the p38 pathway can lead to pathological processes. When the p38 MAPK cascade is activated by pro-inflammatory and stressful stimuli, it can trigger a range of cellular responses, including inflammation, cellular apoptosis, and the inhibition of cell proliferation, differentiation, and invasion [65]. The inhibitory effects of the p38 MAPK pathway on the proliferation, differentiation, and invasion of the HTR8/SVneo trophoblastic cell line have been documented following stimulation with pro-inflammatory cytokines or strong inflammatory activators such as lipopolysaccharide (LPS) [65,66]. These findings are further supported by studies utilizing a rat model of preeclampsia, where LPS, an endotoxin that induces preeclamptic symptoms in Sprague-Dawley rats, activates the p38 pathway through toll-like receptor 4. The placentas of these animals exhibited not only increased p38 activity compared to controls but also displayed consequences of pathway over-activation, including elevated IL-6 levels, impaired trophoblast invasion, and the inadequate remodeling of spiral arteries. Interestingly, inhibiting the p38 pathway alleviated preeclamptic symptoms in LPS-stimulated animals, improving trophoblast invasion and maternal vessel transformation while reducing inflammation in placental tissue [66]. Similar observations related to the upregulation of the p38 pathway have been linked to defects in the placental structure, elevated inflammation, and an increased apoptosis of cells in the junctional zone of rodent placentas, as noted in preeclamptic rats stimulated with N(omega)-nitro-L-arginine methyl ester (L-NAME) to induce clinical symptoms of preeclampsia [67].

Furthermore, immunohistochemical studies of human placentas have also indicated the over-activation of the TLR4/p38 pathway, likely contributing to heightened inflammation [66]. This pathway is also associated with the increased secretion of the sFlt-1 protein from placental tissue; notably, inhibiting p38 in cultured chorionic villi cells reduced the secretion of sFlt-1 into the surrounding environment [68]. Elevated levels of sFlt-1 in maternal blood are recognized as an early indicator of preeclampsia, often appearing several weeks prior to the onset of clinical symptoms [69], and studies have shown that the production of this soluble receptor for pro-angiogenic factors, including PlGF and VEGF, is, additionally, regulated by pathways dependent on JNKs [70].

#### 2.3.4. JNK Pathway

The JNKs pathway belongs to the family of MAPK pathways and is activated by upstream kinases MKK4 and MKK7, which are phosphorylated by various isoforms of MAP3K, including mitogen-activated protein kinase kinase kinase 3 (MAP3K3, also known as MEKK3) and transforming growth factor-beta-activated kinase 1 (TAK1, known as MAP3K7). The engagement of specific MAP3K isoforms for the activation of downstream substrates depends on the stimuli; for example, inflammatory factors such as IL-1 and TNFα, as well as TLR3, TLR4, and TLR9, act through TAK1, while TLR8 activates MEKK3 [71]. However, cytokines and TLR receptors are not the only activators of JNKs, which are represented by three isoforms: JNK1, JNK2, and JNK3. Strong activators of JNKs include UV radiation, DNA-damaging agents, growth factors, and G protein-coupled receptors [62]. Following stimulation, JNKs translocate to the nucleus; however, some part of JNKs remain localized in the cytoplasm, suggesting the presence of cytoplasmic targets for these kinases, such as keratin 8, a protein involved in regulating cell stress and apoptosis [62,65,72]. Although the cytoplasmic substrates for JNKs are still being investigated, the nuclear substrates are well characterized. Among these, the c-Jun transcription factor, a key member of the activator protein-1 (AP-1) complex, is particularly notable; it works alongside c-Fos, which is primarily activated by the p38 pathway, highlighting the crosstalk between the JNK and p38 MAPK pathways [62,73,74].

A study conducted on placental chorionic villi obtained between weeks 5 and 9 of gestation, as well as the HTR8/SVneo trophoblastic cell line, indicated that the JNK/c-Jun pathway is strongly implicated in regulating processes typically disturbed in preeclampsia, such as trophoblast invasion and spiral artery remodeling. The inhibition of the JNK pathway significantly enhanced the invasion efficiency of HTR8/SVneo cells [75]. Furthermore, the JNK pathway, in conjunction with the p38 MAPK pathway, activates apoptosis through the Fas cell surface death receptor (Fas)/Fas ligant (FasL) death system, which is crucial for human embryo implantation [76]. However, evidence suggests that the apoptosis of placental cells in preeclamptic pregnancies also depends on the this death system [77,78]. The cooperation between the p38 and JNK pathways may enhance the activation of common downstream substrates, particularly, transcription factors such as ATF2 and signal transducer and activator of transcription 3 (STAT3) [62,79,80]. Both ATF2 and STAT3 are associated with preeclampsia; studies indicate that elevated levels of ATF2 characterize preeclamptic women, normalizing after low-dose aspirin interventions [81]. Similarly, STAT3 levels are also elevated in placental tissue, but this elevation is, additionally, linked to pathways outside the MAPK family, specifically the JAK/STAT pathway [82].

### 2.4. JAK/STAT Pathways

The protein family of signal transducer and activator of transcription (STAT) functioning as intracellular transcription factors consists of STAT1, STAT2, STAT3, STAT4, STAT5a and 5b, and STAT6 members that, in the JAK/STAT pathway, are activated by Janus family kinases (JAK) represented by Jak1, Jak2, Jak3, and Tyk2 (tyrosine kinase 2) [83,84]. The activated STAT proteins are implicated in numerous cellular processes, including apoptosis, angiogenesis, or the controlling of immunological processes. In non-complicated pregnancy, STAT signaling pathways regulate trophoblast function, supporting the process of migration and invasion, and, additionally, these pathways are responsible for maintaining the balance of immune tolerance, which is essential for the correct course of gestation [85]. Although numerous studies consistently report elevated levels of JAK and STAT proteins in placental samples from preeclamptic pregnancies, animal models of preeclampsia, and trophoblastic cells cultured in environments simulating preeclampsia, the conclusions drawn from these studies are contradictory. Some indicate that the activation of the JAK/STAT pathway in trophoblastic cells supports the development of preeclampsia, driven by immunological processes [86]. The JAK/STAT3 pathway works closely with leukemia inhibitory factor (LIF) to induce inflammation and endothelial dysfunction, characterized by increased levels of intercellular adhesion molecule-1 (ICAM-1) and vascular cell adhesion molecule-1 (VCAM-1), all of which are hallmarks of preeclampsia [87]. Additionally, the detrimental effects of the over-activation of the JAK2/STAT3 pathway have been observed in placentas obtained from animal models of preeclampsia, such as rats stimulated with L-NAME. The inhibition of JAK2/STAT3 regulation through the knockdown of annexin 1 resulted in reduced apoptosis and inflammatory responses in trophoblastic cells derived from L-NAME rats [88]. Conversely, other studies utilizing trophoblastic cell lines emphasize that the strongest activation of STAT1, JAK1/STAT3, and STAT5 pathways in preeclampsia is a compensatory mechanism; these pathways enhance trophoblast proliferation and invasion while exacerbating apoptosis [83,89,90]. These contradictions may arise from the diverse mechanisms of JAK/STAT pathway activation observed in both in vitro and in vivo models, as well as from the various pathways within the JAK/STAT family that have been examined. This complexity highlights the need for a nuanced understanding of how different contexts and stimuli can influence the role of JAK/STAT signaling in preeclampsia.

### 2.5. Nrf2 Signaling Pathway

Nuclear factor erythroid 2-related factor 2 (Nrf2) is a pivotal transcription factor that plays a crucial role in cellular defense against oxidative stress and inflammation, both of which are significantly exacerbated in preeclampsia. Nrf2 is composed of seven highly conserved functional domains, known as Neh1 through Neh7, each serving distinct roles in its regulation and activity. The Neh1 domain facilitates heterodimerization with small Maf proteins, allowing Nrf2 to bind to antioxidant response elements (AREs) in the nucleus, while the Neh2 domain is crucial for interacting with the negative regulator Kelch-like ECH- associated protein 1 (Keap1), thereby mediating Nrf2’s ubiquitination and degradation. The Neh3, Neh4, and Neh5 domains of Nrf2 function as transactivation domains that recruit essential transcriptional machinery for Nrf2’s activity, while the Neh6 and Neh7 domains play role in the inhibition of Nrf2 function; Neh6 enables degradation by binding to β-transducin repeat-containing protein (β-TrCP), and Neh7 supports repression of Nrf2 activity through interaction with retinoic X receptor alpha (RXRα) [91]. Despite the extensive understanding of Nrf2, research findings regarding its localization in non-stressed cells remain inconsistent. Some studies indicate that Nrf2 is predominantly localized in the nucleus [92], where it binds to antioxidant response elements (AREs) in the promoters of various genes. These include those encoding key antioxidant enzymes such as superoxide dismutase (SOD), specifically SOD1 and SOD2, catalase (CAT), glutathione peroxidases (GPx), including GPx1, GPx2, GPx3, and GPx4, and heme oxygenase-1 (HO-1) and other cytoprotective genes [93,94,95]. This is essential for regulating reactive oxygen species (ROS) levels, as optimal ROS levels are vital for maintaining normal cellular signaling and ensuring proper cellular function and communication. In contrast, other studies highlight the cytoplasmic localization of Nrf2, where it associates with its inhibitor, Keap1 [91,96,97]. Under conditions of oxidative stress, characterized by elevated ROS levels, the activity of Keap1 is inhibited, allowing Nrf2 to dissociate from this complex. Once released from Keap1, Nrf2 translocates to the nucleus to regulate the expression of genes involved in antioxidant defense mechanisms.

#### Nrf2 Signaling Pathway in Pregnancy

Interestingly, although preeclampsia is characterized by oxidative stress, in both maternal blood and placental tissue, a high level of ROS is observed; the level of antioxidant enzymes such as HO-1, SOD, GPx, and CAT is depleted [98,99,100,101,102,103]. This depletion may indicate a disturbance in the regulatory mechanisms of ROS by the Nrf2 signaling pathway. Indeed, the hypermethylation of the Nrf2 gene, which inhibits gene expression, is commonly observed in placental tissue from preeclamptic women [104]. However, not all studies report a downregulation of Nrf2; some suggest that in preeclamptic pregnancies complicated by fetal growth restriction (FGR), Nrf2 levels in maternal decidua remain unaffected compared to controls, whereas in preeclamptic pregnancies with normal fetal growth, Nrf2 gene expression is diminished [105]. It is important to note that the liberation of Keap1 from its complex with Nrf2 is not solely dependent on oxidative stress. Evidence suggests that the ERK1/2 signaling pathway plays a significant role in Nrf2 activation in response to VEGF signaling. This mechanism may protect against oxidative stress by promoting the expression of HO-1, which, subsequently, upregulates VEGF expression. In preeclampsia, the reduced activity of the ERK1/2 pathway may contribute to the observed decrease in VEGF bioavailability, potentially exacerbating oxidative damage [106]. Oxidative stress is also a potent inducer of inflammation, and the relationship between the two is reciprocal [107]. Notably, Nrf2 plays a significant role in regulating inflammation, exhibiting the antagonistic properties toward NFκB signaling. Nrf2 binds to the promoter regions of genes encoding pro-inflammatory cytokines, such as IL-6 and IL-1β, which are regulated by NFκB, thereby inhibiting their expression. This interaction highlights Nrf2’s role in modulating inflammatory responses by directly suppressing the transcription of these cytokines. However, NFκB can inhibit Nrf2 gene expression and competes with Nrf2 for binding to the transcriptional coactivator CREB-binding protein (CBP) [108]. Given that NFκB plays a central role in the pathomechanism of preeclampsia, exhibiting over tenfold higher activity in preeclamptic placentas [109], this may further elucidate why the anti-inflammatory and antioxidant mechanisms dependent on Nrf2 are not functioning effectively in this condition.

## 3. Selected Plant Extracts and Their Influence on PE Pathogenesis

Preeclampsia is a disease that occurs in pregnant women; thus, the pharmacologic treatment is limited due to the fact that most of drugs are not recommended for use during pregnancy. The drugs ingested by mother are absorbed in the intestines into the bloodstream, and then, their compounds can act on the fetus through placental blood transfer. When that process takes place in the first or second week of the gestation, it is associated with an ‘all-or-nothing’ phenomenon—drugs can result in fetus death or no adverse outcomes [110]. As the first 12 weeks of pregnancy are crucial for organogenesis (the formation of major body structures) the exposure to drugs in that time can causes teratogenic effects, which are the source of functional or structural dysgenesis of the fetal organs. Administration of drugs after 12 weeks of gestation will not lead to anatomical defects, but to functional ones, such as fetal growth inhibition, developmental disability, and the aggravation of the intrauterine environment, like oligohydramnios [110,111].

Therefore, the use of medications during pregnancy is not always feasible, prompting researchers to increasingly explore natural medicine, particularly plant extracts, for the treatment and prevention of pregnancy-related conditions such as preeclampsia. For centuries, plants have been employed in medicine due to their diverse health properties, including anti-inflammatory, anti-hypertensive, anti-apoptotic, antioxidant, and antiviral effects. However, while plant extracts or their constituents may provide therapeutic benefits for various disorders, it is essential to recognize that the misuse or abuse of these substances can adversely affect fetal development and overall pregnancy outcomes. Research indicates that a significant proportion of pregnant women—up to 80% in certain regions, such as Australia—perceive herbal medicines as safe alternatives to conventional treatments [112]. Nonetheless, some herbal extracts can exhibit cytotoxic effects at elevated concentrations, raising concerns about their safety for both the mother and fetus, particularly when these preparations are consumed without medical supervision alongside prescribed therapeutic agents [113]. Plant extracts can be categorized into two primary groups: primary metabolites, which are vital for the plant’s growth and development, and secondary metabolites, which encompass terpenes, terpenoids, alkaloids, and phenolics—bioactive compounds that enable the plant to adapt to its environment [114]. Various ecological factors—including seasonal fluctuations in temperature and groundwater quality, carbon dioxide concentration, light intensity, ozone levels, soil moisture, soil salinity, and soil fertility—significantly influence the physiological and biochemical responses of medicinal plants. These factors also impact their secondary metabolic processes, thereby altering the chemical constituents of raw plant materials [115,116]. Notably, the composition of secondary metabolites in plants growing within the same environment can vary depending on the specific part of the plant exposed to different environmental elements. Distinct metabolites are synthesized through specific regulatory pathways associated with particular organs, tissues, and cells. Consequently, the composition of secondary metabolites varies not only among different plant species but also according to the specific parts of each plant—including roots and stems, leaves, flowers, fruits, and seeds [117]. Moreover, the methods employed in preparing plant extracts significantly influence their chemical composition. The polarity of the solvent used determines which compounds are extracted; polar solvents are generally more effective for hydrophilic compounds, while non-polar solvents are better suited for lipophilic substances. Additionally, factors such as temperature and extraction duration affect both the yield and stability of bioactive compounds; elevated temperatures may lead to thermal degradation [118,119]. Thus, the selection of solvent and extraction parameters is critical for defining the chemical profiles of plant extracts, which directly influences their efficacy. The following section presents an overview of several significant plant extracts that demonstrate potential in addressing pregnancy-related complications, including preeclampsia.

### 3.1. Astragalus Species

The radix of *Astragalus membranaceus*, and its variety, *Astragalus membranaceus var. mongholicus*, is among the most widely used herbal medicine in the world. This traditional Chinese medicine extract consists of 26 orally available compounds, such as 12 flavonoids, five phenolic acids, five nitrogen-containing compounds, three lignanoids and one coumarin, some of which are presented in the Table 1 [120,121,122]. *Astragali radix* is known for its antioxidant, anti-inflammatory, or immunomodulation properties. Moreover, it seems to improve renal function, enhance cardiac function, decrease blood pressure, dilate blood vessels, and be a potential compound preventing preeclampsia [120,121,122,123]. Zhu et al. tested injections of aqueous extract from *Astragalus* root of radix on Spraque-Dawley rats with L-NAME-induced PE. The results showed that the extract of *Astragalus membranaceus* and *Astragalus membranaceus var. mongholicus* reduces urinary protein and blood pressure levels and improves placental mass. Moreover, it increases the expression of matrix metalloproteinase 9 (MMP-9), which improves placental implantation and blood perfusion [123]. Also, the expression of NFκB, nuclear factor of activated T-cells 5 (NFAT-5), and sFlt-1 in placental tissues was significantly inhibited after *Astragalus* injections treatment. Those findings are very relevant, as MMP-9 can affect the invasive ability of placental trophoblasts, and nuclear transcription factors are crucial for regulating the release of inflammatory factors. Also, both NFκB and MMP-9 play a role in placental vascular remodeling and trophoblast erosion [123]. *Astragalus membranaceus* was also used by Zhao et al. as a plant extract to create a mixture of hydro-alcoholic extracts from four plants: ALAE. ALAE was studied on sows with healthy pregnancies, and its influence on genes related to preeclampsia was tested [120]. The authors suggested that the studied treatment acts on PI3K/Act pathway, and thus, reduces inflammation and apoptosis by decreasing IL-6, IL-1β, and Caspase-3 levels, and increasing IL-10 levels, and improves angiogenesis through increasing MMP-9, peroxisome-proliferator activated receptor gamma (PRARγ), and PlGF levels [120]. Moreover, ALAE seems to improve the placental structure, reduce inflammation, and regulate placental angiogenesis and growth factor receptors, and thus, to increase the total number of born piglets, as well as live and healthy ones. Preeclampsia is, among other features, characterized by increased levels of NFκB, sFlt-1 and pro-inflammatory cytokines. Hypertension and proteinuria are also characteristic features of that disease. Increased blood pressure and abnormal immune response in preeclamptic pregnancies are the cause of improper placentation, and thus, a reduction in the number of live and healthy offspring and their weight gain. The above findings indicate that *Astragalus* species extract treatment improves all those abnormalities, suggesting its beneficial effects in potential PE treatment. Moreover, the studies on *Astragalus membranaceus* show that its extract acts on MAPK pathway through decreasing the level of p38 in macrophages [124] and IPEC-J2 (intestinal porcine enterocytes isolated from the jejunum of a neonatal unsuckled piglet) cells [125]. Additionally, studies on liver have demonstrated that *Astragalus membranaceus* decreases the level of NFκB, indicating that it acts on NFκB pathway [126]. The inhibitory effect on NFκB activity may also be influenced by the inhibition of p53, a protein involved in the preeclamptic pathway of NFκB activation. Indeed, oxidative stress and p53 protein levels were found to be reduced in endothelial cells isolated from rats treated with polysaccharides extracted from *Astragalus* [127]. Additionally, *Astragalus membranaceus* has been shown to influence gut microbiota composition in experimental rats, specifically affecting the *Firmicutes/Bacteroidetes* (F/B) ratio and reducing the abundance of *Clostridia* and *Proteobacteria*. These alterations suggest that *Astragalus* not only impacts inflammatory pathways but also modulates gut health, potentially enhancing its therapeutic effects in preeclampsia [126].

### 3.2. Eucommia ulmoides Oliver

Another plant used in traditional Chinese medicine is *Eucommia ulmoides Oliver* (EU), also known as Du-Zhong, Mumian, Gutta-percha tree, Silk Cotton Skin, Silk Pulled Skin, Sixian, Sizhong, and Sijinshu. Extracts from EU are usually made from the dried bark and are believed to prevent miscarriages, as well as to be beneficial for the liver, kidneys, bones, and muscles. Moreover, EU seems to lower blood pressure, regulate glucose and lipid metabolism, prevent osteoporosis, and have antioxidative and immunomodulatory effects [128,129]. *Eucommia ulmoides Oliver* extract consists of numerous compounds, such as flavonoids, lignans, steroids, iridoids, phenylpropanoids, terpenoids, and polysaccharides. A total of 246 components have been isolated from this plant, some of which are shown in Table 1 [128,129,130]. L. Zhao et al. used *Eucommia ulmoides Oliver* as one of the selected plant hydro-alcoholic extracts to create an ALAE mixture. As it was previously said, ALAE treatment is thought to act through the PI3K/Act pathway, reducing inflammation and apoptosis, and enhancing angiogenesis [120]. The positive effects of ALAE treatment on the placenta and the fetuses may be caused by the actions of EU, as it is known for its antihypertensive, antioxidative, and immunomodulatory properties. Because of those effects, the development of the placenta and the angiogenesis process can be improved. It is known that in preeclamptic pregnancies, the placentation and angiogenesis processes are disturbed, and thus, the number of born offspring, including live and healthy ones, is decreased in comparison to healthy pregnancies. Additionally, the effects of *Eucommia ulmoides Oliver* were tested on liver cells, and the results indicated that extract of *Eucommia ulmoides Oliver* increases Nrf2 and decreases JNK levels [131]. Moreover, the studies on microglial cells showed that *Eucommia ulmoides Oliver* elevates Nrf2 and IκBα/β levels, and reduces NF- κB, p38 and JNK levels [132]. Therefore, the *Eucommia ulmoides Oliver* may support the treatment or prevention of conditions associated with inflammation and oxidative stress. Moreover, *Eucommia ulmoides Oliver* has prebiotic potential and may improve gastrointestinal health [133].

### 3.3. Euterpe oleracea Mart.

*Euterpe oleracea Mart.*, also known as ‘*açaí*’, is popular due to its anti-inflammatory, antioxidant, and neuroprotective properties, among others. Extract of *açaí* seeds is rich in catechin, epicatechin, ferulic acid, and vanilic acid, as shown in Table 1 [134,135]. Silva et al. tested *açaí* seeds hydro-alcoholic extract (ASE) on Wistar rats with L-NAME-induced PE. The results indicated the cardiovascular effects of ASE treatment, as it reduced hypertension [134]. Additionally, antioxidant effects were described, as the level of lipid peroxidation products was decreased. The treatment with *Euterpe oleracea Mart.* seeds extract also improved microalbuminuria and the weight gain of fetuses, and the mass gain of placentas [134]. The impact of *Euterpe oleracea Mart.* on the placentation process was not studied; however, there are studies showing the influence of *açaí* on the molecular pathways involved in placentation. The research on human endothelial cells demonstrated that *Euterpe oleracea Mart.* extract acts on the Nrf2 and MAPK pathways through elevating Nrf2 and ERK1/2 levels [136]. While direct studies on the placentation process are limited, the described findings highlight *açaí’s* potential to modulate the pathways essential for placental health, suggesting applications in reproductive medicine. Future research is needed to confirm these effects and explore the therapeutic benefits of *Euterpe oleracea Mart.* extract in placental development and pregnancy outcomes.

### 3.4. Moringa oleifera

One of the most widely studied plants is *Moringa oleifera*, also called horseradish tree, drumstick tree, mother’s best friend, or miracle tree. Its many parts are used for nutritional and medicinal purposes, as it is known, among other properties, for its antioxidant, anti-inflammatory, anti-hypertensive, antifungal, and antibacterial effects [137]. The therapeutic benefits of *Moringa oleifera* drew attention to the closer study of this plant and led to the discovery of its composition. Some of the *Moringa oleifera* extract compounds are shown in Table 1. Batmomolin et al. investigated the effects of *Moringa oleifera* leaves hydro-alcoholic extract (EMOL) on Wistar rats with L-NAME-induced PE. They found that EMOL decreases the serum level of pro-inflammatory IL-17 and sFlt-1, and also reduces hypertension in PE rat model. The authors suggest that the anti-inflammatory properties of EMOL are the results of regulating the NFκB pathway [138]. Additionally, the studies on *Moringa oleifera* have demonstrated that its extract decreases the level of NF-κB in kidney tissue [139], the JNK level in femoral tissues [140], and JAK2 and STAT3 levels in colon tissues [141]. These findings suggest that *Moringa oleifera* extract may have anti-inflammatory and antihypertensive effects, potentially being beneficial for placentation process by modulating key signaling pathways such as NFκB, MAPK, and JAK/STAT. Further research could explore the therapeutic potential of *Moringa oleifera* in managing inflammatory conditions and hypertension in preeclamptic women.

### 3.5. Uncaria rhynchophylla

Another herb from traditional Chinese medicine is *Uncaria rhynchophylla*, also known as *Gou-teng*. It has anti-hypertensive, anti-epilepsy, anti-inflammatory, and neuroprotective effects. In China, *Uncaria rhynchophylla* is used to treat various disorders, such as hypertension, epilepsy, and even preeclampsia [142,143,144]. It also helps to treat fever, dizziness, nervous disorder, and cerebrovascular diseases. *Uncaria rhynchophylla* extract consists inter alia of flavonoids, pentacyclic triterpene esters, and indole alkaloids, some of which are presented in Table 1 [142,143,144]. The influence of *Uncaria rhynchophylla* was tested by Wu et al. on Sprague-Dawley rats with (lipopolysaccharide) LPS-induced PE. Their results indicated that the hydro-alcoholic extract treatment decreased systolic blood pressure, ameliorated proteinuria levels and increased fetal weight, placental weight, and the number of live fetuses. Moreover, the levels of pro-inflammatory cytokines, like IL-6, IL-1β, TNFα, and interferon gamma (IFN-γ), were decreased in serum and the placenta. Additionally, the authors showed that *Uncaria rhynchophylla* extract treatment decreased the level of NFκB in the placenta [143]. The high level of NFκB in PE women causes the increase in pro-inflammatory cytokine levels and the decrease in anti-inflammatory ones. In consequence, an inflammatory state occurs, contributing to endothelial dysfunction [145,146]. Furthermore, the effects of *Uncaria rhynchophylla* extract on molecular pathways were studied on macrophages stimulated with LPS and the hippocampus, and the results showed that *Uncaria rhynchophylla* acts on the MAPK pathway through the reduction in p38 and JNK [147,148]. Therefore, *Uncaria rhynchophylla* extract may affect the mentioned pathways in placental cells. However, further studies are needed to confirm this hypothesis. Interestingly, some in vivo studies indicate that *Uncaria rhynchophylla* presents the impact on the composition of gut microbiota [149,150].

### 3.6. Vitis labrusca

Special attention should be also paid to the species of grapevines *Vitis labrusca*, as it seems to have anti-hypertensive, anti-inflammatory, and antioxidant properties. Additionally, it shows cardioprotective, renoprotective, hepatoprotective, and neuroprotective effects. As the *Vitis labrusca* grape skin extracts have been widely studied, their compounds are determined and some of the main active compounds are listed in Table 1 [151,152]. According to the in vivo study conducted on Wistar rats with L-NAME-induced PE, *Vitis labrusca* grape skin extract administration led to a reduction in insulin resistance and hypertension. Moreover, the treatment with *Vitis labrusca* grape skin extract increased the number of alive fetuses [153]. De Moura et al. conducted an experiment to investigate the effects of a hydro-alcoholic extract of *Vitis labrusca* on Wistar rats with L-NAME-induced preeclampsia, confirming its anti-hypertensive properties. Additionally, their results showed that the weight loss of dams treated with extract was significantly smaller than in animals treated with L-NAME without extract treatment, and the blood vessels in mesenteric vascular bed were significantly dilated after treatment [154]. Preeclampsia is associated with placental hypoxia, which causes the narrowing of the blood vessels of placenta. This, in turn, leads to decreased blood flow and insufficient nutrient supply to the fetus. In consequence, the fetus’s weight is reduced. Moreover, the research on *Vitis labrusca* extract indicates that it decreases the NFκB level in thoracic aorta tissue [146], human monocytic U937, and human umbilical vein endothelial cells (HUVEC) [145], and thus, regulates the NFκB pathway. Additionally, *Vitis labrusca* acts on the MAPK pathway through increasing the ERK1/ERK2 levels [155].

*Vitis labrusca* grape skin extract seems to have antihypertensive, anti-inflammatory, and antioxidant properties, and it has been shown that this extract can modulate the key pathways involved in placentation. This makes *Vitis labrusca* a promising candidate for further studies on the effect of *Vitis labrusca* extract on the efficacy of treatment or reducing the risk of preeclampsia.

To sum up, plant extracts seem to have great potential to support the processes of preeclampsia treatment and prevention. They improve the pathophysiological processes occurring in preeclampsia, underlying hypertension, proteinuria, and inflammation. Moreover, studies have demonstrated that plant extracts positively influence fetal and placental weight, as well as the survival rates of offspring. However, the difficulties in obtaining a constant composition of active substances in plant extracts from the same plant species make researchers focus on isolating individual substances from plants and studying their influence on PE processes. These composite differences may result from the region in which the plant is grown or the method of extract preparation. Furthermore, the testing of pure compounds allows for the precise evaluation of their biological activity, enabling the determination of whether a specific molecule exhibits pharmacological effects or not [156]. Analyzing Table 1 reveals that several compounds commonly present in the extracts are recognized as potential agents against preeclampsia. This observation highlights the importance of these compounds in developing therapeutic strategies for managing preeclampsia. Consequently, further research is crucial to determine whether these agents could serve as effective treatments or prevention methods for this condition. This inquiry remains open and warrants careful consideration in future studies, and it will be further discussed in the subsequent sections of this manuscript.

**Table 1:** Commonly reported compounds found in analyzed plant extracts.

Plant Extract Compounds	*Astragalus* *species*	*Eucommia* *ulmoides Oliv*	*Euterpe* *oleracea*	*Moringa* *oleifera*	*Uncaria* *rhynchophylla*	*Vitis* *labrusca*
anthocyanins	-	-	-	-	-	+
**ascorbic acid**	-	+	-	+	-	-
**caffeic acid**	+	+	-	+	-	+
**catechin**	-	+	+	-	+	-
cyanidin	-	-	-	-	-	+
**epicatechin**	-	+	+	+	+	+
**ferulic acid**	+	-	+	-	-	-
**gallic acid**	-	+	-	+	-	+
hirsuteine	-	-	-	-	+	-
isorhynchophylline	-	-	-	-	+	-
**kaempferol**	+	+	-	+	-	+
maldivin	-	-	-	-	-	+
n-octacosanoic acid	-	+	-	-	-	-
petunidin	-	-	-	-	-	+
**quercetin**	+	+	-	+	-	+
resveratrol	-	-	-	-	-	+
rhynchophylline	-	-	-	-	+	-
**rutin**	+	+	-	+	+	-
**vanilic acid**	+	+	+	-	-	-
yohimbine	-	-	-	-	+	-
	[121,122]	[128]	[134]	[157]	[144]	[158]

Legend: (+) indicates the presence of selected plant compounds in the described plant extracts. (“-”) indicates that the compound is absent or not reported in the literature; however, “not reported” does not imply that this secondary metabolite is absent from the extracts. The composition of plant extracts can vary due to environmental conditions, plant parts used, and extraction methods, as previously discussed. Compounds that occur most frequently among the analyzed extracts are presented in bold. The last row of Table 1 includes literature references in brackets.

## 4. Selected Chemical Compounds and Their Influence on PE Pathogenesis

### 4.1. Caffeic Acid

One of the most commonly isolated chemical compounds found in the described extracts is caffeic acid. This hydroxycinnamic acid belongs to the phenolic molecules and occurs in various vegetables, fruits, seasonings, and beverages. It has a beneficial impact on porcine oocyte maturation, fertilization rates, and blastocyst formation in matured oocytes. Moreover, it inhibits the oxidative damage in HUVECs and increases antioxidant effects in endometrial cells. In the in vitro research on HTR8/SVneo cells, treatment with caffeic acid significantly attenuated DNA damage and apoptosis after H_2_O_2_ exposure [159]. In preeclampsia, the structural modifications of the umbilical vein endothelium and a decreased vasodilation of endothelium have been observed. Moreover, the plasma of preeclamptic women contains increased levels of different endothelial activation markers, such as cytokines or anti-angiogenic factors (sFlt-1). All the above leads to oxidative stress, which causes further endothelial damage. The elevated level of oxidative stress in preeclamptic placenta cells increases DNA fragmentation and can lead to trophoblasts apoptosis [160]. One scientific report indicates that caffeic acid influences the PI3K/Act, Nrf2, and NFκB molecular pathways by increasing the levels of Nrf2 and PI3K/Act while decreasing the level of NFκB [161]. The modulation of these critical pathways suggests that caffeic acid may play a significant role in mitigating the pathophysiological processes associated with preeclampsia. Consequently, the use of this acid in the prevention or treatment of preeclampsia could be advantageous in reducing oxidative stress and inflammation.

### 4.2. Epicatechin

Epicatechin is one of the flavonoids abundant in pome fruits, tea, cocoa-based food, and grapes. It plays a role in increasing endothelium-dependent vasodilation and decreasing systemic blood pressure and platelet activation, among other effects [162]. Kluknavsky et al. performed an in vivo study on a borderline-hypertensive rat model. The rats were treated with epicatechin and the results showed that epicatechin decreased systolic blood pressure [163]. Other research has demonstrated that epicatechin influences molecular pathways, revealing a decrease in the levels of NFκB, p38, and JNK [164]. These findings suggest that epicatechin may contribute to cardiovascular health by promoting vasodilation and reducing blood pressure through its impact on molecular pathways. Its ability to decrease the levels of NFκB, p38, and JNK indicates its anti-inflammatory potential, making it a promising candidate for managing hypertension and related inflammatory conditions.

### 4.3. Ferulic Acid

Ferulic acid, (E)-3-(4-hydroxy-3-methoxy-phenyl)prop2-enoic acid, is a hydroxycinnamic acid occurring in plants. It catalyzes the formation of phenoxy radicals and, thus, protects cell integrity. Moreover, it inhibits the expression of IL-6 by reducing the activity of NFκB through the down-phosphorylation of IκB Kinase Complex [165]. Ferulic acid also improves inflammation through the regulation of TNFα, IL-6, and IL-1β secretion and prevents apoptosis as well as cell damage caused by oxidative stress and inflammation. Chen et al. studied the ferulic acid influence on Sprague-Dawley rats with L-NAME-induced PE. The results indicate that the treatment improved blood pressure and decreased urine protein and urinary volume [165]. Additionally, it increased anti-inflammatory factors, such as IL-4 and IL-10, and decreased inflammatory factors, such as TNFα, IL-6, and IL-1β, NFκB p65, and PlGF. Moreover, the treatment decreased Bax expression and increased Bcl-2 expression [165]. Bax protein is responsible for stimulation of cell apoptosis, and Bcl-2 protein prevents cell death. Maintaining the balance between pro-apoptotic and anti-apoptotic proteins during pregnancy is crucial for the proper tolerance of the maternal immune response, remodeling of spiral arteries, differentiation of villous trophoblast, and turnover of trophoblast. However, the increased level of Bax, decreased level of Bcl-2, and higher apoptosis rate of trophoblastic cells are observed in PE. As the ferulic acid reduces the levels of NFκB and pro-inflammatory cytokines and Bax expression, it may be an important substance in PE treatment. Additionally, the influence of ferulic acid on molecular pathway was tested on adipocytes, and it occurs that treatment with ferulic acid decreases the JNK level, as well as reducing the level of NFκB [166]. Furthermore, studies conducted on liver homogenates have demonstrated that ferulic acid not only reduces the level of NFκB but also, simultaneously, elevates Nrf2 level. This dual action suggests that ferulic acid may play a significant role in modulating inflammatory responses and enhancing cellular antioxidant defenses, highlighting its potential therapeutic benefits in conditions characterized by oxidative stress and inflammation, such as preeclampsia [167].

### 4.4. Quercetin

Quercetin is a flavonol being found in dill, oregano, onions, citrus fruits, apples, berries, green tea, green leafy vegetables, seeds, nuts, broccoli, olive oil, and grapes. It has anti-inflammatory, antioxidant, antihypertensive, antidiabetic, antiatherosclerotic, antiobesity, antihypercholesterolemic, and anticancer properties [168,169,170]. Li et al. studied the effects of quercetin on Sprague-Dawley rats with LPS-induced PE. They found that quercetin treatment significantly reduces systolic blood pressure and decreases the level of albumin in urine [168]. Moreover, the treatment improved abnormal uteroplacental angiogenic status through decreasing the sFlt-1/PlGF ratio and the expression of inflammatory cytokines, such as IL-6 and TNFα. Quercetin treatment also improved the weight and size of the fetuses, and weight of the placenta in preeclamptic rats [168]. However, Tanir et al., in their research on Sprague-Dawley rats with L-NAME-induced PE, did not confirm that quercetin treatment reduces blood pressure. Nevertheless, they showed that the proteinuria and a high death rate of offspring was decreased after the treatment [169]. These discrepancies may be due to different mechanisms inducing hypertension in model animals. LPS mainly acts by inducing inflammation, and L-NAME by abolishing the vasodilatory properties of the endothelium [171,172]. Moreover, L-NAME can induce hypertension in non-pregnant animals, whereas LPS can only induce hypertension in pregnant animals, so the LPS model is closer to the course of PE.

The molecular mechanism of quercetin treatment was studied by Wang et al. on the HTR-8/SVneo cell line, and the results show that quercetin strongly binds with AKT1 and MMP-9, increasing their expression. The study also proved the anti-inflammatory and anti-apoptotic effects of the treatment, which were confirmed by the decreased expression levels of TNFα, IL-6, IL-1β, Bax, and Caspase-3 [170]. The MMP-9 level is elevated in women in healthy pregnancies. It plays a crucial role in trophoblast invasion into the decidual stroma. However, the level of MMP-9 in preeclamptic women is decreased, which disturbs the process of the proper implantation of trophoblastic cells. Moreover, the studies on thymocytes and splenocytes indicate that quercetin reduces the levels of NFκB and IκBα, thereby influencing the NFκB signaling pathway. Additionally, this compound demonstrates inhibitory properties toward the JAK/STAT pathway [173]. These findings highlight quercetin’s therapeutic potential, particularly for managing conditions like preeclampsia due to its anti-inflammatory, antihypertensive, and antioxidant effects. Interestingly, quercetin also modulates gut microbiota composition [174].

### 4.5. Other Important Compounds

In addition to the compounds previously listed in Table 1, several other substances are frequently discussed in the literature regarding the treatment of preeclampsia, making their influence on the molecular characteristics of the disease worthy of consideration. Among these are curcumin, resveratrol, and puerarin.

#### 4.5.1. Curcumin

Curcumin, a polyphenol compound, is found in the rhizomes of *Curcuma longa*. It is known to have anti-inflammatory, anti-apoptotic, antioxidant, antihyperlipidemic, antiviral, anticoagulant, and antitumor properties; thus, it has been used for medical purposes for years [175,176,177]. Gong et al. carried out the in vivo study on Sprague-Dawley rats with LPS-induced PE. Their results indicate that curcumin treatment reverses the shallow placental implantation caused by LPS. As mentioned before, shallow invasion is observed in preeclamptic pregnancies and is associated with improper vessel transformation. This results in narrow structures, which makes the delivery of oxygen and nutrients to the fetus and placenta much more difficult. Moreover, curcumin seems to increase the weight of the fetuses and be effective in improving renal damage induced by LPS [175]. In the study conducted by Gong et al., the expression levels of TLR4, NFκB, and inflammatory cytokines such as IL-6 and Monocyte Chemotactic Protein 1 (MCP-1) were increased by LPS; however, those expressions were decreased after the curcumin treatment. Therefore, the authors suggest that the anti-inflammatory effects of curcumin may be attributed to its regulation of the TLR4/NFκB pathway [175]. The influence of TLR4 is particularly significant in the context of preeclampsia. Romão-Veiga et al. conducted studies on peripheral blood mononuclear cells (PBMCs) isolated from women with preeclampsia and healthy controls. Their results demonstrated that TLR4 expression is significantly higher in the plasma of preeclamptic women compared to those with healthy pregnancies, potentially creating a cytokine environment that is unfavorable for pregnancy [178].

Zhou et al., in their in vivo study on C57/B6 mice with LPS-induced PE, showed that the anti-inflammatory properties of curcumin are confirmed by decreasing TNFα, IL-1β, and IL-6 expression levels and increasing the phosphorylated Akt levels. The authors suggest that the anti-inflammatory properties of curcumin may result from suppressing NFκB [177]. The inhibitory effect on NFκB may arise from the inhibition of classical NFκB activators or from curcumin’s negative impact on p53, a protein implicated in the preeclamptic pathway of NFκB activation in placental cells [13,177,179]. Additionally, Zhou et al. reported that curcumin treatment resulted in decreased blood pressure and proteinuria in LPS + curcumin-treated mice, along with reduced inflammation. These effects may emerge from the upregulation of Akt phosphorylation as a consequence of curcumin’s action [177].

Qi et al. studied the effect of curcumin on the HTR-8/SVneo cell line, which is a human trophoblastic cell line presenting the features of the first trimester placenta. The in vitro model was exposed to oxidative stress, and then, treated with curcumin. The results showed that curcumin protects cells from apoptosis induced by oxidative stress, by decreasing the expression level of cleaved-Caspase 3 and by the improvement of the Bcl–2/Bax ratio [176]. Curcumin also seems to reduce accumulation of oxygen free radicals through activation Nrf2 signaling pathway [176]. This suggests that it may be one of the multiple mechanisms through which curcumin exerts its effects on the regulation of molecular pathways potentially implicated in the pathomechanisms of preeclampsia. Additionally, the influence of curcumin on molecular pathways was studied on colon cells, and the results showed that curcumin acts on MAPK pathway through a reduction in the p38 level [180].

Studies also demonstrated that curcumin seems to have a regulatory influence on intestinal flora, including the diversity, abundance, and constitution of microorganisms [181].

#### 4.5.2. Resveratrol

Another widely studied compound is resveratrol (RSV). This polyphenolic compound has been primarily found in grapes, peanuts, berries, and apples. It helps regulate blood pressure, inflammation, and oxidative stress, among other effects. RSV offers various biological and therapeutic benefits, including cardiovascular protection, anti-hypertensive benefits, anti-atherosclerotic properties, protection for the liver, anti-cancer effects, support for endothelial function, anti-thrombotic and anti-angiogenic effects, lipid regulation, mitochondrial biogenesis, and anti-diabetic effects [182,183,184]. All of which make it a potential candidate for PE treatment.

Zou et al. conducted studies on both in vitro and in vivo models. The results of their on HTR-8/SVneo cell line suggest that resveratrol protects trophoblastic cells against apoptosis and inhibits the level of oxidative stress [182]. The in vivo study on Wistar albino rats with L-NAME-induced PE shows that RSV decreases blood pressure in preeclamptic rats [4,182]. Bueno-Pereira et al. carried out a study on cells derived from umbilical arteries from pregnant preeclamptic women and HUVECs treated with resveratrol [184]. The results showed that RSV regulates oxidative stress through the L-arginine–NO pathway by increasing NO bioavailability and decreasing arginase activity in endothelial cells. Moreover, in endothelial cells and umbilical arteries, resveratrol downregulated endothelial dysfunction genes, such as Caspase-3, ICAM-1, and von Willebrand factor (vWF) [184]. Reducing ICAM-1 levels through resveratrol is critical, as ICAM-1 is a leukocyte adhesion molecule expressed on endothelial cells, with increased levels observed in preeclamptic patients. Elevated ICAM-1 expression promotes leukocyte adhesion and the production of reactive oxygen species (ROS), ultimately leading to endothelial damage. Furthermore, resveratrol’s protective effects against DNA damage, inflammation, cellular senescence, and apoptosis may be mediated through the AMPK/ERK5 signaling pathway. Notably, resveratrol has been demonstrated to enhance the expression of antioxidant enzymes in endothelial cells via the activation of ERK5 [185]. However, the influence of resveratrol on the molecular pathways in placentas was not examined. However, research not specifically related to pregnancy indicates that resveratrol influences the MAPK pathways by reducing the levels of p38 and JNK [186]. Additionally, a study involving a murine macrophage cell line cultured with LPS indicates that resveratrol inhibits the activity of NFκB and IκBα, thereby impacting the NFκB signaling pathway. Furthermore, resveratrol was found to decrease STAT3 levels, suggesting its inhibitory effect on the JAK/STAT pathway [187]. As resveratrol has been shown to enhance apoptosis rates, reduce oxidative stress, and alleviate endothelial dysfunction while influencing pathways associated with placentation, it merits further investigation into its potential benefits for the treatment or prevention of preeclampsia.

#### 4.5.3. Puerarin

*Pueraria lobata*, in traditional Chinese medicine, is widely known as *Gegen* and puerarin (Pue) is one of the main active ingredients extracted from its root. Puerarin has anti-inflammatory, cardioprotective, vasodilative, neuroprotective, and antitumor properties [188].

Liang et al. studied puerarin treatment in both in vitro (HTR-8/SVneo cell line) and in vivo (Sprague-Dawley rats with LPS-induced PE) PE models [188]. The study shows that LPS increases placental expression levels of sFlt-1, tissue plasminogen activator (tPA), endothelin 1 (ET-1), Bax, Caspase-3, Caspase-8, Caspase-9, TNFα, IL-6, and IFN-γ levels, but also decreases the Bcl-2 expression level and IL-4 level in placentas. Moreover, LPS causes placental changes such as necrotic and inflammatory cell infiltration changes. The results of the study indicated that puerarin treatment reverses or improves all of the above pathological changes caused by LPS. LPS also changes the expression of genes involved in inflammatory pathways, such as NFκB, TNFα, and IL-17, cytokine–cytokine receptor interaction, and apoptosis. Pue treatment suggested that it has a protective effect in PE models regulating inflammatory responses, significantly reducing hypertension and proteinuria and improving abnormal pregnancy outcomes [188]. He et al. tested the antioxidant properties of puerarin on the HTR-8/SVneo cell line, which was treated with H_2_O_2_ in order to mimic oxidative stress-induced trophoblast cell injury.

Moreover, the studies on vascular smooth muscle cells, fibroblasts, and hepatic cells indicate that the puerarin acts on MAPK pathways through a reduction in p38 and JNK levels [189,190] but also on the Nrf2 pathway through increasing the Nrf2 level [190,191,192]. These findings suggest that puerarin may be effective in managing preeclampsia by reducing inflammation, oxidative stress, and cellular apoptosis in placental tissues. Due to puerarin’s potential, its extract may support the effect of treatment or prevention for preeclampsia and related pregnancy complications.

#### 4.5.4. Rutin, Ascorbic Acid, Gallic Acid, Kaempferol, and Vanillic Acid

Some of the compounds bolded in the Table 1 have not been studied in the context of preeclampsia. Despite lack of the direct connection between those compounds and PE, it cannot be omitted that they are involved in the regulation of many molecular processes underlying PE development.

One of the compounds of special interest is rutin. Its effects are well described in cardiovascular disorders. Rutin removes ROS, but also activates Nrf2, which protects cells against oxidative stress [193,194,195,196]. Moreover, rutin plays an important role in decreasing the rate of apoptosis, by increasing the Bcl–2/Bax ratio and reducing the Caspase-3 level [197,198]. Rutin also shows anti-inflammatory properties, through preventing the activation of NFκB and decreasing levels of NFκB and pro-inflammatory cytokines, such as TNFα and IL-6 [180,182,183]. Additionally, rutin seems to help in proper angiogenesis, protecting against impaired arteriolar vasorelaxation [194,196]. The literature reports that rutin has an influence on the molecular pathways associated with placentation. First of all, the studies on liver homogenate and fibroblasts show that rutin also elevates the level of Nrf2, another key molecule of the molecular pathway characteristic for placentation [199,200]. Moreover, the studies on the hippocampus indicate that rutin acts on the MAPK pathways through increasing the ERK1/ERK2 ratio [201]. Additionally, other research proved that treatment with rutin decreases the levels of STAT3, JNK, and p38 [202].

Another antioxidant substance is ascorbic acid, known as vitamin C, also related to proper blood pressure [203,204,205,206]. Rutin and ascorbic acid are combined to enhance the anti-inflammatory, anti-apoptotic, and anti-oxidative properties of both substances [207,208,209]. The studies on the brain tissue demonstrated that ascorbic acid decreases levels of p38, and thus, acts on the MAPK pathway, crucial for correct placentation [210].

Gallic acid appears to reduce hypertension, oxidative stress, and inflammation by decreasing the levels of TNFα, IL-1β, IL-6, and MCP-1 in cardiovascular disorders [211,212,213]. Research conducted on liver cells indicates that gallic acid increases the levels of Nrf2 and ERK1/ERK2 [214]. Furthermore, studies involving a human colon carcinoma cell line (Caco-2) incubated with LPS demonstrated that gallic acid decreases levels of p38 and JNK [215]. Additionally, gallic acid influences the PI3K/Akt pathway by increasing PI3K/Act levels in human alveolar basal epithelial cells (A549) [216]. Investigations on lung tissue have shown that gallic acid affects both the Nrf2 and NFκB pathways through the elevation of Nrf2 levels and the reduction of NFκB levels [217]. Therefore, it seems that gallic acid could be helpful in improving the placentation process in PE.

Further, the influence of kaempferol and vanillic acid have been studied on preeclamptic models—Sprague-Dawley or Wistar rats with LPS administration. Both substances seem to show cardioprotective and antioxidative effects and reduce blood pressure. Kaempferol also reduces the levels of pro-inflammatory cytokines, such as IL-6 and TNFα [218,219]. Studies on human Jurkat T leukemia and lung cells demonstrated that kaempferol acts on the NF- κB pathway through decreasing the NFκB level [220,221] but also on the MAPK pathway through reducing p38 and JNK levels [221]. Additionally, studies on vanillic acid indicate that it acts on the MAPK pathway through decreasing p38 and JNK levels in chondrocytes [222], but also through increasing the ERK1/ERK2 levels in pancreatic β-cells [223]. Moreover, vanillic acid increases the Nrf2 level in HUVECs, and, thus, affects the Nrf2 pathway [224].

The evidence presented above shows that both extracts and their components, by regulating molecular pathways in placental cells, can be considered as alternative methods supporting the process of preventing or treating preeclampsia. These compounds also have a modulatory effect on the maternal microbiome by participating in the microbiota–gut–placenta axis.

## 5. The Microbiome and Its Influence on Preeclampsia

As mentioned before, the specificity of the state of pregnancy forces researchers and physicians to search for non-invasive therapeutic approaches. In addition to the natural compounds, mainly derived from the traditional Chinese and Eastern medicine, current science is gradually stressing the importance of the human microbiome and its changes occurring through the pregnancy and pregnancy-related disorders. This area seems especially promising considering the fact that many of the mentioned natural compounds (e.g., curcumin or quercetin) were proved to exert a positive influence on microorganisms known to enhance healthy pregnancy development [225]. Recently, more and more studies are directed towards understanding the placental microbiota and the utilization of this knowledge in the future therapies.

### 5.1. Changes in the Gut Microbiome in Preeclampsia

During pregnancy, the maternal organism must undergo various physiological changes to ensure optimal conditions for the developing fetus. Among these, gradually gaining scientific recognition, are modifications in microflora of the expectant mother. The human microbiota consists of approximately 100 trillion organisms, mostly inhabiting the digestive tract [226]. Some scientists debate the possible existence of a microbiome of placental origin—that theory, however, is supplanted, among others, by the theories of oral or vaginal translocation, in which the presence of the bacteria in placenta is explained by the microorganisms ascending from other locations [227,228,229]. Despite the controversies and the lack of evidence on its local function, the presence of microorganisms in the placenta is confirmed, as is the fact that it does not lead to negative pregnancy outcomes [227,228,230].

On the other hand, the abundance of scientific reports with regard to gut microbiota confirms its role in food processing (e.g., segregating food substances such as vitamins), xenobiotics detoxification, and maintaining gut integrity with the epithelium renewal, thus affecting the immune system [226]. In case of pregnancy, the gut microbiome modifies through the time, differing between each trimester (T1, T2, T3), allowing the fetus to develop [231]. In the phylum level, among the most prominent categories of bacteria in pregnant women (both healthy and PE) are *Firmicutes*, *Actinobacteria*, *Bacteroidetes*, *Proteobacteria*, and *Fusobateria* [231,232] and the ratio of *Firmicutes* to *Bacteroidetes* (F/B) stands as an important parameter reflecting the possible malfunctions of microbiota [233].

Any imbalance in the composition of intestinal microbiota may result in systemic inflammation and metabolic abnormalities, thus influencing the development of numerous illnesses. Regarding pregnancy, bacterial imbalance has recently been suggested to have a possible impact on preeclampsia development. Majority of the studies indicate that, in PE, among the main bacteria phylum, there is a significant decrease in the abundance of *Firmicutes* (including *Akkermansia*) and *Bacteroidetes*, while the amount of *Proteobacteria* and *Fusobacteria* is elevated [232,234]. The reduced abundance of *Akkermansia* in PE patients was also negatively correlated with blood pressure and urine protein [235]. In contrast, during healthy pregnancy, *Akkermansia* and *Firmicutes* bacteria are known to be increased, having a protective effect on mother and the fetus [226,231]. The majority of the studies stays in accordance with the described dependence, yet some of them provide data indicating an increased relative abundance of *Bacteroidetes*, especially during T3 [234,236]. An increase in this class of gram-negative bacteria, together with the decrease in the amount of the phylum of *Firmicutes*, was even described as a taxonomic biomarker of PE in T3 [236].

One of the key characteristics of PE is excessive inflammation. Both *Proteobacteria* and *Fusobacteria*, abundant in PE, belong to gram-negative bacteria, which contribute to the larger proportion of LPS presence. LPS is a gram-negative bacteria product enhancing local and systemic inflammation [232]. Its presence in PE gut microbiota imbalance contributes to the disrupted integrity of the epithelium and facilitates LPS inflammatory actions, which, in PE, are mediated through the TLR4 pathway [175]. Thus, it is possible that the gut microbiota imbalance can lead to excessive pathogenic infection, triggering pro-inflammatory immune responses, including complement activation and cytokines release, leading to PE development. The fact that LPS itself is used in vivo to exert a PE-like environment in animal models seems to support the above theory [237,238].

Further, according to the Zhao et al. research team, the *Firmicutes* to *Bacteroidetes* (F/B) ratio is significantly higher in the PE group compared to healthy controls (*p* < 0.001) [232]. A similar tendency is also observed both in animals and humans affected by obesity or hypertension [239,240,241].

### 5.2. The Influence of Microbiota Imbalance on Placental Development

Although the exact pathomechanism of PE is still vague, it is commonly assumed that the central organ involved in observed changes is placenta. In humans, the placenta is formed within the first three months of pregnancy and then grows parallel to the developing uterus. It consists of parenchyma, the chorion and amnion, and the umbilical cord [242]. Its maternal part comes from the endometrium and is called decidua, while the fetal parts develop from the chorionic sac derived from the zygote. Generally, after the fertilization, the morula evolves from the fertilized ovum and, later, develops into the embryo and fetal placenta. The outer cell mass differentiates into the trophoblast, which, upon contact with the endometrium, initiates the development of two distinct layers: the syncytiotrophoblast and the cytotrophoblast. These layers are essential for forming the fetal side of the placenta, specifically, the chorionic villi. Additionally, a portion of the cytotrophoblastic cells transforms into extravillous trophoblast (EVT), which plays a vital role in the remodeling of maternal blood vessels. This transformation is essential for establishing proper blood flow to support fetal development [242,243]. In contrast, rodents do not develop placental villi. Instead, the fetal portion of their placenta consists of two layers: the labyrinth, which corresponds to the human villous part, and the junctional zone, which serves as a source of invasive trophoblast [238]. As in PE, the process of placentation is disrupted, and maternal vessels fail to remodel, the placental bed remains poorly perfused, and the fetus develops in the state of hypoxia and excessive inflammation. Hence, no effective treatment is known to cure or prevent the disease; ongoing studies are focused on searching new possible therapies to help women with PE.

**Figure 1:**
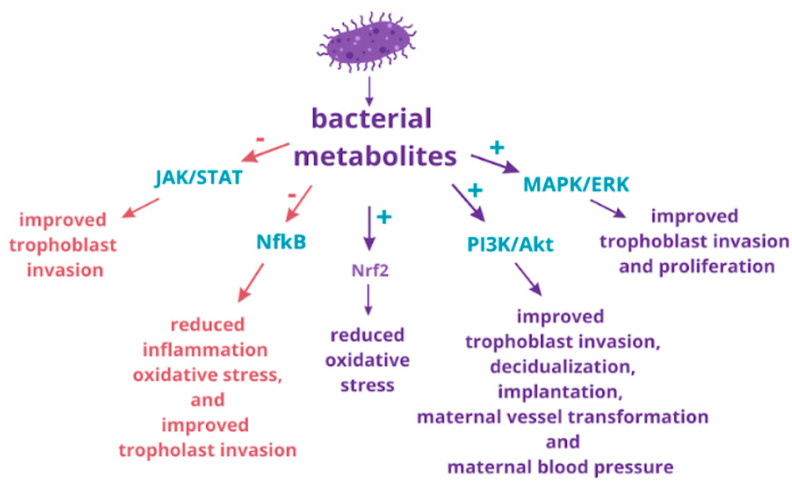
Main molecular targets of the selected bacterial metabolites. Key molecular pathways influenced by microbiota are marked with turquoise color. The dark blue color is reserved for pathways and targets activated by bacterial metabolites, and the red one for the targets being inhibited. Legends: + positive, i.e., activating impact of bacterial metabolites on studied pathway; “-” negative, i.e., inhibiting impact of bacterial metabolites on studied pathway.

It is currently being speculated whether microbiota imbalance can influence the risk of developing PE. Although scientists argue about the origin of the microorganisms in placenta, it cannot be denied that this compartment is no longer believed to be sterile [230]. Recently, an in vivo study conducted with the use of germ-free (GF) pregnant mice and one with a depleted maternal gut microbiome (treated with broad-spectrum antibiotics; ABX) revealed a possible connection between presence of microbiota and placental development [244]. The studied groups exhibited a lower placental weight at embryonic day 14.5 (E14.5) in comparison to the control. Further, micro-computed tomography of the derived placentas showed significant reductions in the total placental volume, together with reduced volume and tissue density, in particular, in the placental labyrinth subregion [244]. Therefore, it was hypothesized that maternal microbiota changes can alter the development of feto-placental vasculature, further negatively influencing gas and nutrients supply, which is a key characteristic of PE pathogenesis. The main molecular targets of bacterial metabolites are presented in Figure 1.

As it was mentioned before, the microbiome can influence human organisms in many ways, for instance, by regulating circulating metabolites [226]. Following this lead, the research group of G.N. Pronovost et al. considered the role of the microbial generation of short-chain fatty acids (SCFAs) in regulating placental development [244]. SCFAs (such as acetate, propionate, and butyrate) are produced by bacterial carbohydrate fermentation and are significantly reduced in the maternal serum of microbiota-deficient dams [245]. Further, other animal studies demonstrated the positive influence of SCFAs on placental integrity, including spiral artery remodeling and reduced inflammation, among other effects, through NFκB inhibition [246,247]. Studies conducted with the stool samples of almost 200 pregnant women (including healthy and PE ones), and following in vivo and in vitro functional experiments, confirmed PE gut microbiota dysbiosis and revealed significant reductions in SCFA-producing bacteria as well as SCFAs itself [235]. According to the authors, propionate/butyrate (and, as also mentioned previously, *Akkermansia*, SCFAs-producing bacteria) significantly ameliorated symptoms in preeclamptic rats by promoting the polarization of M2 macrophages in the placental bed. Additionally, propionate was shown to promote trophoblast invasion.

### 5.3. Microbiota and Molecular Pathways of Pregnancy

Among the many molecular pathways implicated in the PE pathogenesis are the JAK-STAT signaling pathway, NFκB signaling pathway, PI3K/Akt signaling pathway, and MAPKs signaling pathway [248]. Each of them is involved in some of the processes regulating placental development, like trophoblast cell proliferation, migration, and fusion, and is highly controlled by numerous factors, including cytokines, growth factors, and hormones.

One of the key characteristics of PE is shallow placentation. The in vitro study conducted on the HTR-8/SVneo cell line, corresponding to the first trimester trophoblast, revealed that IFN-γ, increased in PE and released, among other mechanisms, by decidual natural killer (NK) cells, activates the JAK/STAT1 pathway, reducing cell invasion [249]. On the other hand, a study conducted by Jin, et al. indicated that one of the *Akkermansia* metabolites—propionate—significantly reduced STAT1 levels, which was accompanied by a downregulation of iNOS (inducible nitric oxide synthase) and TNFα protein, both known to be implicated in PE pathogenesis [235,248,250].

Recent studies confirmed that, although NFκB level is higher in PE patients, most of the NFκB activation pathways are downregulated, and the alternative pathway responsible for its activation in trophoblastic cells may involve the p53/RSK1 complex [13]. Additionally, one of the receptors involved in NFκB activation is TLR4. Women with PE are known to express higher TLR4 and NFκB levels [251]. Further, TLR4 was reported to increase p53 activity [252]. Therefore, it is possible that changes in the TLR4/p53/NFκB axis may be involved in PE pathogenesis. In the in vitro study conducted with the HTR-8/SVneo cell line, some characteristics of PE, like the upregulated expression of TLR4, pro-inflammatory (TNFα), and antiangiogenic (endoglin; ENG/sENG) factors, were evoked by LPS stimulation [235]. The above effects were significantly attenuated by bacterial propionate. At the same time, propionate upregulated the expression of matrix metalloproteinases (MMP-2 and MMP-9), which play a key role in trophoblast invasion [235]. An additional factor known to be connected with NFκB is Nrf2 [240]. As mentioned before, its role is critical in protecting cells from oxidative stress, a function that, in PE, may be disrupted due to the lower Nrf2 levels in PE patients. Interestingly, the known probiotic *Lactobacillus rhamnosus* GG (LGG) was shown to activate Nrf2 in both flies and mice, modulating its susceptibility to oxidative injury [253].

During healthy pregnancy, the PI3K/Akt signaling pathway is known to influence, among other processes, decidualization, implantation, cell growth, proliferation, migration, and survival [248]. In PE patients, it is generally believed to be downregulated [254]. According to the in vitro experiments on the HTR-8/SVneo cell line, propionate significantly increases the p-Akt/Akt ratio, activating the Akt signaling through G protein-coupled receptor 41 (GPR41) and, therefore, promoting trophoblast invasion [235]. The protective role of propionate, regarding PE development, also confirms the fact that activated GPR41 was previously reported to decrease the blood pressure in mice [255].

The MAPKs signaling pathway is mainly involved in trophoblast invasion, fusion, and differentiation; at the same time, MAP/ERK molecules are associated with pathways related to PE endothelial dysfunction and immunological alterations [248]. An in vivo study conducted with GF, SPF (specific pathogen-free), and BIF (GF colonized with *Bifidobacterium breve*) mice revealed that a lack of maternal gut microbiota significantly impaired the growth of the placental labyrinth zone, including a reduced placental surface area and fetal capillary volume [256]. These changes were, however, partially restored by *Bifidobacterium breve* administration. Further, the quantification of the expression of selected genes in the labyrinth zone revealed an altered pattern mainly for the MAPK pathway. The Mapk1 level was shown to be higher in both the GF and BIF groups, when compared to SPF controls. At the same time, on the protein level, ERK expression was lower, but only in the GF group, suggesting the influence of *Bifidobacterium breve* on MAPK signaling. Additionally, *B breve* exposure increased the mRNA level of p38MAPK, confirming its possible involvement in the regulation of the pathways related to placental development [256]. Additionally, another MAPKs signaling pathway member—ERK1—was shown to be influenced by an *Akkermansia* metabolite—indole-3-propionic acid (IPA) [257]. According to the outcomes of the in vivo experiment conducted on mice, IPA significantly increased ERK1 phosphorylation, leading to its activation [108]. The above is especially important considering the influence of ERK1, among other factors, on placental cell differentiation.

### 5.4. Probiotics in PE Prevention

Considering the described influence of microbiota on placentation and vascularization during pregnancy, it seems that probiotics supplementation may be beneficial to the women suffering from PE. Yet, studies do not stay in agreement regarding this issue. A recently conducted meta-analysis encompassed 29 trials with the use of different probiotics in PE patients [258]. The studied probiotics were from the group of *Lactobacillus*, *Bifidobacterium*, *Streptococcus*, and *Propionibacterium*. Four of the selected trials used vaginal probiotic capsules or vaginal yogurts, while other probiotics were administered orally. This systematic review led the authors to the conclusion that the tested probiotics may have no effect on the risk of PE, preterm birth, gestational age at birth, gestational hypertension, and gestational diabetes [258]. However, the authors stress the limitations of their study, which included the small size and heterogeneity of the studied population. In contrast to the described results are studies confirming significant gut dysbiosis in PE women [234] and the influence of probiotics on placental cells, TLR signaling, and cytokine release [259,260]. Moreover, in the case of probiotic supplementation, the F/B ratio should be considered when choosing the proper treatment, as the probiotics of choice will differ depending on the ratio [261]. In addition to the mentioned-earlier positive effects of *Akkermansia*, its product, propionate, and *Bifidobacterium*, some articles report the beneficial effects of *Lactobacillus* strains, which were shown to interact with placental cells [262]. The study conducted on the purified trophoblast cells examined the role of heat-killed *Lactobacillus rhamnosus* GG (LGG) [263]. The outcome of the study confirms its beneficial effects, as LGG stimulation resulted in a higher expression of anti-inflammatory cytokines IL-4 and IL-10. Additionally, in cells treated with LPS, LGG stimulation reversed LPS-induced TNFα release, with no change in hormone release (e.g., progesterone) [263]. Taken together, despite the evident limitations of the described studies (which mainly include an insufficient sample size), the presented results suggest that probiotics may serve as modulatory agents in the human placenta without altering basic trophoblast functions. This fact, together with the ability of probiotics supplementation to reduce blood pressure in humans [264], allows to assume its future application in diseases like PE.

## 6. Conclusions

**Figure 2:**
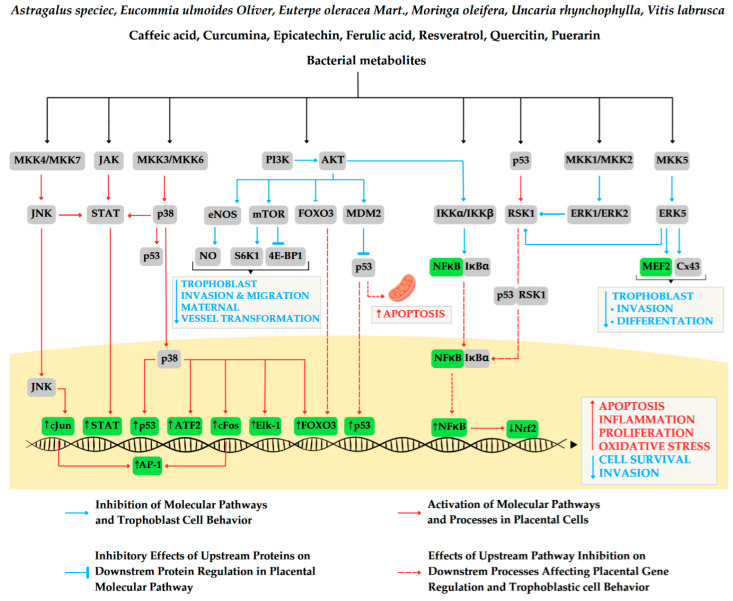
Dysregulated placental molecular pathways in preeclampsia: consequences and connections to nutraceutical interventions. Green squares represent key transcription factors modulated by placental molecular pathways: 4E-BP1, eukaryotic translation initiation factor 4E-binding protein 1; AP-1, activator protein-1; ATF2—activating transcription factor 2; cFos,—Fos proto-oncogene; cJun,—Jun proto-oncogene; c-Jun—Jun proto-oncogene; MEF2—myocyte enhancer factor-2; Elk-1—transcription factor ELK1; FOXO3—forkhead box O3; IKKα—inhibitory-κB kinase alpha; IKKβ—inhibitory-κB kinase beta; mTOR—mammalian target of rapamycin; NFκB—nuclear factor kappa B; Nrf2—Nuclear factor erythroid 2-related factor 2; p53—protein 53; STAT—signal transducer and activator of transcription. Gray squares represent key proteins involved in molecular pathways: AKT—protein kinase B; Cx43—connexin-43; eNOS—endothelial nitric oxide synthase; ERK—extracellular signal-regulated kinase; IκBα—inhibitor of nuclear factor kappa B; JAK—Janus family kinases; MDM2—mouse double minute 2 homolog; MKK—mitogen-activated protein kinase kinase; NO—nitric oxide; p38—protein 38; PI3K—phosphatidylinositide 3-kinase; RSK—ribosomal protein S6 kinase alpha-1; S6K1—p70 ribosomal protein S6 kinase 1.

The dysregulation of molecular pathways in trophoblastic cells can significantly influence cellular behavior, including trophoblast proliferation, migration, and invasion potential, ultimately affecting the remodeling of maternal blood vessels (Figure 2). These factors are intricately linked to the pathophysiology of preeclampsia. Consequently, harnessing the potential of plant extracts and their bioactive compounds to rectify the dysregulated mechanisms in placental tissue emerges as a promising avenue for developing novel natural therapies for conditions such as preeclampsia. There is a growing body of evidence indicating that specific bioactive compounds found in various plant extracts can provide beneficial effects in reducing the risk of preeclampsia and improving therapeutic outcomes. By enhancing antioxidant defenses, reducing inflammation, and promoting processes essential for proper placentation—including trophoblast invasion and maternal vessel transformation—these natural products may offer complementary strategies alongside conventional therapies. A similar impact on pregnancy development may also be observed with a broader category of nutraceuticals, such as probiotics, which improve maternal microbiota and serve as a source of beneficial metabolites. The maternal microbiome plays a crucial role in influencing pregnancy outcomes; the metabolites secreted by microorganisms are recognized not only as regulators of molecular pathways, including those in the placenta, but also as modulators of maternal immunological processes that support the necessary immunosuppression for healthy pregnancy development. Interestingly, some nutraceuticals may work synergistically; for instance, specific bioactive compounds from plant extracts can enhance the growth of beneficial gut bacteria, leading to an increase in the production of metabolites that further support placental health and fetal development. Therefore, it is worthwhile to explore whether combining these plant-derived bioactive compounds with probiotics could create a powerful strategy for optimizing maternal health during pregnancy, potentially reducing the risk of complications such as preeclampsia. This integrated approach may harness the complementary effects of both types of nutraceuticals, promoting a healthier microbiome and improving overall pregnancy outcomes.

## Data Availability

Not applicable.
